# Biophysical Parameters Modification Could Overcome Essential Hearing Gaps

**DOI:** 10.1371/journal.pcbi.1000161

**Published:** 2008-08-29

**Authors:** A. Kern, C. Heid, W.-H. Steeb, N. Stoop, R. Stoop

**Affiliations:** 1 Institute of Neuroinformatics, University and Swiss Federal Institute of Technology Zurich, Zürich, Switzerland; 2 International School of Scientific Computing, University of Johannesburg Auckland, South Africa; 3 Computational Physics, Swiss Federal Institute of Technology Zurich, Zürich, Switzerland; Istituto Nazionale di Ottica Applicata, Italy

## Abstract

A majority of hearing defects are due to malfunction of the outer hair cells (OHCs), those cells within the mammalian hearing sensor (the cochlea) that provide an active amplification of the incoming signal. Malformation of the hearing sensor, ototoxic drugs, acoustical trauma, infections, or the effect of aging affect often a whole frequency interval, which leads to a substantial loss of speech intelligibility. Using an energy-based biophysical model of the passive cochlea, we obtain an explicit description of the dependence of the tonotopic map on the biophysical parameters of the cochlea. Our findings indicate the possibility that by suitable local modifications of the biophysical parameters by microsurgery, even very salient gaps of the tonotopic map could be bridged.

## Author Summary

The cochlea, the mammalian hearing sensor, is a formidable biophysical construct in many respects. Its task is to pick up environmental auditory information, which provides us with a sensory communication channel without which we experience great problems in our every day life. In its extreme form, the lack of hearing capability often leads to social isolation. Mending hearing deficits—increasingly important in societies of growing average age—is difficult, not least because of a delicate interplay between the brain and the sensor. Here, we investigate to what extent the hearing sensor could be tuned in such a way that regions of malfunction are circumvented by relaying the signal to areas of normal functionality. The means by which we envisage achieving this goal is through local changes of the biophysical parameters of the cochlea. By investigation of a detailed biophysical model of the cochlea, we find that nature indeed appears to offer such a possibility.

## Introduction

**Figure 1:**
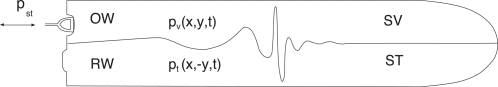
Uncoiled cochlea. Emergence of a travelling wave on the BM due to a sinusoidally varying sound wave with stapes amplitude *p_st_* (OW: oval window, RW: round window, ST: scala tympani, SV: scala vestibuli).

The heart of the mammalian auditory system is the hearing sensor, the cochlea. Acoustic signals arriving in the form of sound pressure waves are funneled by the outer ear towards the tympanic membrane, forcing the latter to oscillate. These oscillations are transmitted to the oval window, a membrane-covered oval opening to the cochlea, by the middle ear ossicles. Within the cochlea, the oscillating membrane elicits incompressible and inviscid hydrodynamic surface waves, comprising the basilar membrane (BM) that partitions the tube containing the cochlear fluid (see Figure 1).

**Figure 2:**
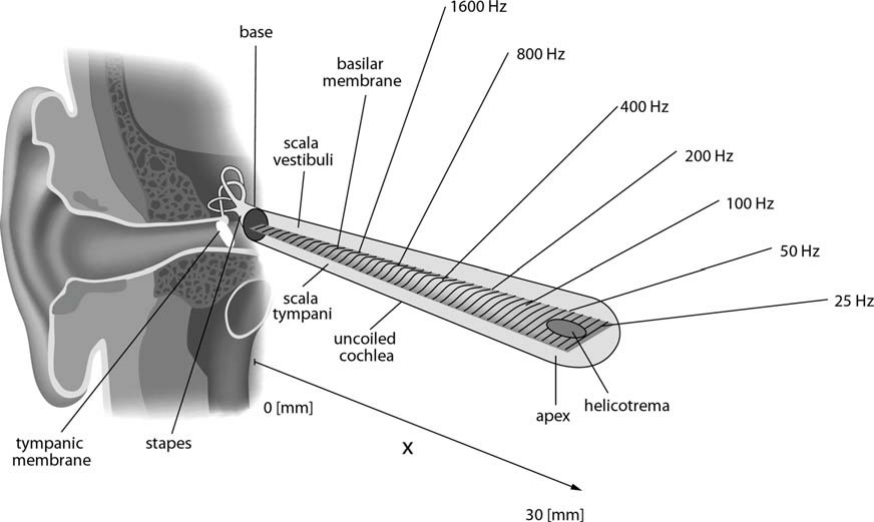
Uncoiled cochlea with BM. The position *x* of the maximal amplitude of the travelling wave corresponds in a 1-to-1 way to a stimulus frequency. (Adapted from [26].)

Using coordinate *x* to express the distance from the oval window towards the apex, the BM transversal stiffness *E* has the dependence *E*(*x*) = *E*_0_ e^−*αx*^, where *α*≈3 cm^−1^ [1]. The generated travelling waves [2] display space-dependent amplitudes that increase from base towards the apex, until they attain their maxima at frequency-dependent locations, *x_c_*(*ω*). It can be shown [1],[3] that as *x* approaches *x_c_*(*ω*), the wave stalls, i.e., *v_G_* = 0, and that the wave number *k*(*x*,*ω*) diverges. Beyond *x_c_*(*ω*), strong attenuation suppresses further travelling. Hence, the location of maximal wave amplitude encodes the stimulus frequency in a 1-to-1 way (the “tonotopic map” (TM), see Figure 2). Along with the BM, the organ of Corti with the attached outer hair cells (OHC) oscillates. Cells located at distance *x* provide an active amplification most effective for waves oscillating at the cell's preferred, or characteristic, frequency *ω_c_*(*x*) [4],[5].

Measured OHC-amplification profiles can be modelled by driven Hopf oscillators [3],[6]. It is known that systems closely below a Hopf bifurcation act like small-signal amplifiers [7],[8]. The amount of amplification is determined by the closeness of the wave frequency to the local system's preferred frequency *ω_c_*(*x*) on one hand, and the local system's distance to the Hopf bifurcation point on the other hand (the closer to the bifurcation point, the stronger the amplification). A biophysiologically detailed model of the cochlea based on these insights [9] has reliably reproduced the salient physiological measurements of the mammalian cochlea [3], [10]–[12]. An electronic implementation using a slightly modified coupling among the active elements [13], reproduces the physiological measurements to the extent that it is hard to distinguish between the physiological and the electronic outputs [14]. The theoretical model, the electronic implementation, and corresponding physiological evidence suggest that the closeness to the bifurcation point can be controlled via a cortico-cochlear feedback loop. By means of this mechanism, the set of the active amplifiers (hair cells) can be tuned towards the amplification of a predetermined signal shape, i.e., towards an auditory object. The degeneration of the hair cells that provide the active amplification, is the most common origin of hearing deficits, where often whole bands of OHC are affected. Because of the importance of the cortico-cochlear hearing loop, the caused hearing impairment cannot be overcome by current hearing aids technology.

In this contribution, we investigate on biophysical grounds whether the TM could theoretically be modified in order to overcome this problem, by a modification of the biophysical parameters of the cochlea. In particular, we study whether modifications can be identified that send incoming frequency information originally targeted to handicapped OHC, to regions of intact OHC. In this case, all salient properties of the cochlea, such as its high input-dependent sensitivity, two-tone-suppression, combination-tone-generation and the ability to tune in into sound sources, would be preserved, and could be used for information processing. It is the aim of this contribution to show that by suitable alterations of the biophysical parameters of the cochlea, such a remapping is, in principle, possible.

## Results

We use an energy-based passive modeling approach that, on the desired level of description, has previously reliably described the physical processes within the cochlea [3], [10]–[12]. The full details of this approach are given in the Materials and Methods section. In short, this approach departs from the energy balance equation [15],
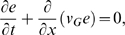
(1)where *e*(*x*,*ω*) denotes the one-dimensional energy density pertaining to a traveling wave, propagating with group velocity *v_G_*(*x*,*ω*) and oscillating with frequency *ω*. The equation is valid both for linear and nonlinear waves and allows us to include in a simple way external power sources, denoted by *a*(*x*,*e*,*ω*), representing the amplification by outer hair cells. From Equation 1 we obtain the change of the energy contained between points *x*_1_,*x*_2_ of the cochlear duct as
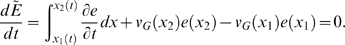
(2)In this equation, the first term denotes energy supplied into or dissipated from the interval [*x*_1_(*t*),*x*_2_(*t*)], while the last two terms take care of the total energy leaving the interval. In the steady state situation, the contributions must compensate. From this, we deduce that
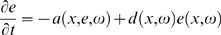
(3)holds, where *d*(*x*,*ω*) denotes the rate of viscous dissipation. Inserting this expression into the energy balance Equation 1, we obtain the *cochlea model differential equation* [3]

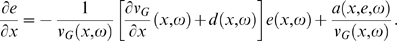
(4)Traditional cochlear models generally lead to partial differential equations, involving often detailed assumptions about geometry and forces within the cochlear canal. In contrast, our energy-based approach leads to an ordinary differential equation. This allows us to separate between the passive components of the cochlea on the one hand and the active amplifiers on other hand.

From the equipartition theorem, we can express the traveling wave amplitude *A*(*x*,*ω*) by the wave energy density *e*(*x*,*ω*) and the BM stiffness *E*(*x*),
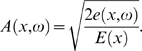
(5)Denoting the amplitude of the stapes displacement by *A*_0_, the passive traveling wave amplitude has the expression
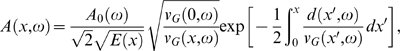
(6)where *A*_0_(*ω*) : = *ωA*_0_.

**Figure 3:**
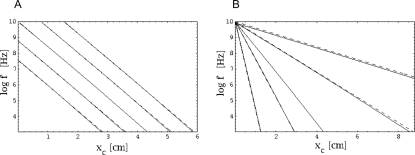
(a) *E*(*x*) = *E*_0_ e^−*αg*(*x*)^. Analytical prediction (dashed) together with the corresponding numerical evaluation (solid) for space dependence a) *g*(*x*) = *x* ^2^ and b) *g*(*x*) = 2 ln(*x*+1), respectively. For comparison, the full straight line displays the unmodified TM. (b) *E*(*x*) = *E*_0_′ e^−*α*′*g*(*x*)^, using *E*_0_′ = 10^2^ *E*_0_ and *α*′ = 3*α*. Analytical prediction (dashed), together with the corresponding numerical evaluation (solid) for the space dependence a) *g*(*x*) = *x* ^2^ and b) *g*(*x*) = 2 ln(*x*+1), respectively. For comparison, the full straight line displays the unmodified TM.

Based on a two-dimensional surface-wave analogy of the cochlea [16], the group velocity of the travelling wave *v_G_*(*x*,*ω*) has the form
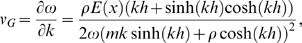
(7)where *ρ* is the cochlear fluid density, *m* the BM mass density, and *h* the diameter of the cochlear canal. The wave number *k*(*x*,*ω*) obeys the dispersion relation
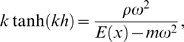
(8)whereas the dissipation rate *d*(*x*,*ω*) can be expressed by [9]

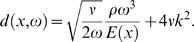
(9)In this equation originally due to Lighthill [17], the last term, which describes the viscous losses by internal friction among the fluid elements, is dominant. *ν* denotes the cochlear fluid kinematic viscosity. The locus of maximal travelling wave amplitude, associated with a frequency *ω*, is therefore related in a 1-to-1 way with a characteristic place *x_c_*(*ω*), which defines the TM. Numerical evaluation of the TM *f*(*x*) from Equation 6, using natural parameters, reveals a linear semilogarithmic behavior of the form (see Figure 3)
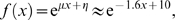
(10)where the obtained values result from a least-squares-fit (mean absolute error 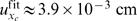, mean absolute error deviation 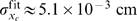).

In order to estimate the effects introduced by changed biophysical parameters, the space derivative of the travelling wave amplitude, 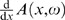 needs to be evaluated. We are interested in the behavior in the close neighborhood of the peak, where *k* becomes exceedingly large, so that *kh*≫1 (the short-wave limit). In this limit, an approximative relation emerges that is sufficiently accurate and manageable that has the form
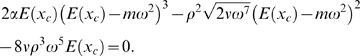
(11)


This equation provides the desired description of the impact of parameter variations to the TM. We are unaware that with classical, not explicitly energy-based approaches [18],[19], a similar characterization could be achieved.

Whereas Equation 11 is strongly nonlinear in the space coordinate *x*, the effects by variations of *m*, *ν*, and *ρ* on the TM are amenable to a simple qualitative discussion nevertheless. Using the substitution Δ : = *E*(*x_c_*)−*mω* ^2^, for *x*≤*x_c_*(*ω*), Equation 11 can be written as
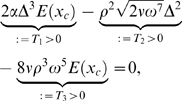
where the transversal stiffness *E*(*x_c_*), *mω* ^2^, and *E*(*x_c_*)−*mω* ^2^ are positive (otherwise for any value of *x*, Equation 11 would not hold). *E*(*x_c_*) and *mω* ^2^ are of the same order of magnitude. For Equation 11 to hold, *T*_1_ needs to balance the two subtractive terms *T*_2_ and *T*_3_. Owing to the different powers associated with the parameters in *T*_1,2,3_, the TM is affected more by changes in *m* and *ρ* than by changes in *ν*. The following theorem can be seen as the direct benefit of our energy-based description, where a proof is provided in the Materials and Methods section.

### Theorem

(I) Space-dependent or -independent increase/decrease of *ρ*, *ν*, and *m* result in left-/right-shifts of the TM.

(II) A change of the transversal stiffness exponent from *αx* into *α*′*g*(*x*′), with *g*(*x*) invertible but otherwise arbitrary, affects the TM as follows (with *η* as in Equation 10):
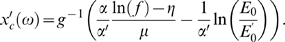
(12)


**Figure 4:**
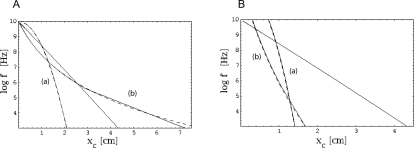
(a) 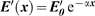. Analytical prediction (dashed) and corresponding numerical evaluation (solid). From left bottom to right top: 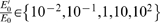. (b) *E*′(*x*) = *E*_0_ e^−*α*′*x*^. Analytical prediction (dashed) together with the corresponding numerical evaluation (solid). From right top to left bottom: 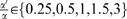.

TM obtained from variations of BM transversal stiffness, evaluated numerically from Equation 6 and predicted analytically by Equation 12, are compared in Figure 4. Taking as the reference the numerical evaluations of the TM from Equation 6, the approximation Equation 11 predicts the effects on the TM up to a numerical accuracy of ∼1.5×10^−2^ cm.

Changes in the initial transversal stiffness *E*_0_ lead to a shift of the TM parallel to the original TM (see Figure 4a). Variations in the decay constant *α* result in a rotation of the TM around the intersection of the original TM with the frequency axis (Figure 4b). Alterations of the space-dependence of the exponent, i.e., *x*→*g*(*x*), result in a non-linear space-dependence of the TM in a semilogarithmic plot (see Figure 3).

These observations are sufficient to construct TM profiles according to will and need. In particular, by suitable modifications of the transversal stiffness (or the surface tension *T*∼(*x*), which in the domain of interest are phenomenologically related by *T*∼(*x*) = *FE*(*x*), with *F*≈10^−5^ *m* ^2^ [9]), using Part II of the theorem, the TM can be forged in such a way that large intervals of hair-cell dysfunction are bridged.

**Figure 5:**
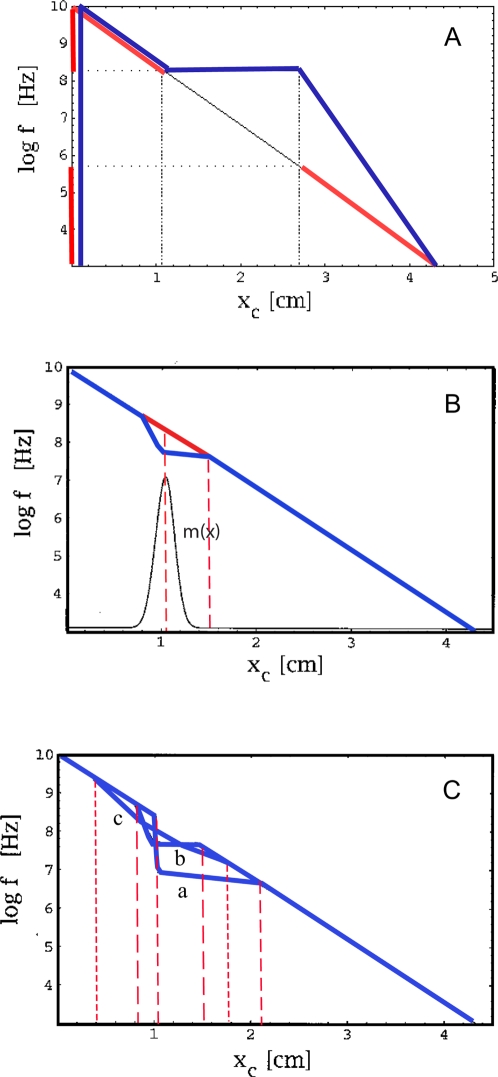
(a) TM constructed by discontinuous variation of the stiffness parameters *α*, *E*_0_, constructed according to part II of the theorem. Dotted lines indicate the defect region in frequency space (*f*∈[300,4000] Hz), dash-dotted lines the corresponding cochlea area. Red: TM before, blue: TM after remapping. (b) Bridging gaps by an additional Gaussian mass distribution 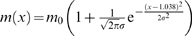, where *σ* = 0.1. Red: Relevant part of TM before, blue: TM after remapping. (c) Effects by additional Gaussian mass distributions of *σ*: *σ*∈{0.01,0.1,1} (graphs a–c, respectively). The dashed lines indicate the essentially modified areas. The achieved space shifts on the TM are obtained by horizontals leading from the modified to the original TM.

Assume, as an example, that the outer hair cells are damaged across the frequency range *f*∈[300,4000] Hz. Such a handicap across the essential frequency band of human speech would result in a severe hearing deficit. By using Equation 12, the space-dependence of transversal stiffness could be modified in such a way that the obtained piecewise continuous TM maps this frequency interval into a region of intact hair cells. The modified TM, as calculated numerically or analytically, demonstrates that the procedure serves the purpose perfectly (Figure 5a).

As a second example, we consider how a local variation of the mass of the BM can be used to bridge gaps. Suppose that the mass distribution is modified from Π_0_(*x*) to 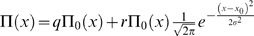. According to the first part of the theorem, *q*≤1 determines the initial shift of the TM. Whereas such a shift may be important for medical applications, for the present example we will not need it, hence we choose *q* = 1. By the second term, we model the attachment of an additional, Gaussian distributed mass distribution of strength *r*, centered around *x*_0_ (Figure 5b). Although in this case the theorem makes no explicit quantitative predictions, it correctly predicts that as *σ* is decreased and the distribution thus becomes more localized, the compressed part and the bridging interval become more prominent, but at the cost of an increased deviation from the desired horizontal orientation of the curve (Figure 5c). Outside the bridged intervals, the modified TM essentially coincide with the original one, even though Π(*x*)>Π_0_(*x*), ∀*x*. According to our theorem, a modification of the remaining biophysical parameters leads to qualitatively similar modifications of the TM. Whereas the observed effects appear overall strongest and best realizable in the case of a modification of the mass density, a modification of other parameters as well as multiple locations of modifications, could be considered in order to achieve optimized results.

## Discussion

Our study is based on a passive cochlea model that has already reliably served as the blueprint for a biomorphic cochlea model and its electronic implementation [3], [9]–[13]. Using the short-wave approximation as the key tool, we have analytically, and quantitatively, predicted the effects of variations of BM mass density, fluid viscosity and fluid density on the TM. We have been able to analytically predict the effects of variations of the transversal stiffness on the passive TM. The obtained insights can be used as a benchmark and guideline for the effects by different kinds of cochlear malformation.

Moreover, a paradigm has been derived that allows one to evaluate the modifications of the local transversal stiffness, mass density, fluid density viscosity, required to bridge defective regions on the BM. We provide here but an short overview of why we believe that the obtained results could have medical relevance. An in-depth discussion of this topic would clearly lead beyond the scope of this research. Although the working BM is extremely sharply tuned, where this tuning comes from is still a matter of debate [20]. In particular, it is as yet unknown to what extent the frequency tuning is due to the BM and to what extent it is an intrinsic property of the active amplifiers, the OHC. There is evidence that the passive BM is not as broadly tuned as was initially suspected [20],[21] and that isolated mammalian OHC are not sharply tuned as initially thought [21],[22]. It is conceivable that it is only in their (nonlinear) combination that these two subsystems achieve the remarkable sharp tuning property. Finally, the efferent connections to the OHC are also able to influence the tuning. We believe that due to the plasticity of the auditory cortex, these OHC could nevertheless amplify frequencies arriving at the remapped places, at least in moderate cases of remapping. Which of the identified changes of the biophysical parameters could be engineered and, in particular, what difficulties will be encountered in a surgical application of the developed framework, remains to be seen. It should, however, be kept in mind that most routine surgery of our days was barely imaginable even a few decades ago. To which extent the auditory cortex would find it easy to deal with an altered TM (proper co-operation of the active elements and successful processing by higher auditory centers), is another question. This issue, however, appears to be softened by the extraordinary plasticity of the human brain, which is already exploited in current cochlear implant technology. For any further investigations into these directions, the obtained results can serve as valuable benchmarks and guidelines.

## Materials and Methods

### Classical Cochlea Modeling

One of the classical references for modeling the passive membrane behavior is Zwislocki's seminal paper [23], where he analyzed an electrical transmission line model of the cochlea originally proposed by Wegel and Lane in 1924 [24]. Peterson and Bogert [25] presented the first mathematical analysis of a hydrodynamical cochlea model. In later works (see, e.g., [1]), these classical models of the cochlea were greatly elaborated. Zweig [18] later deduced the BM transfer function from actual measurements. Moreover, he was able to demonstrate that the organ of Corti actively enhances the cochlear traveling wave. Lighthill [19] analyzed the classical hydrodynamical cochlea models from the point of view of energy propagation, but did not develop a proper wave-energy-propagation model of the cochlea.

### Details of Our Energy-Based Cochlea Modeling

A model that achieved this and accordingly allowed to include active amplification by outer hair cells in a transparent way was introduced in [3] (see also [9] for details). This model departs from the energy balance equation [15],
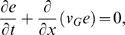
(13)where *e*(*x*,*ω*) denotes the one-dimensional energy density pertaining to a traveling wave propagating with group velocity *v_G_*(*x*,*ω*) and oscillating with frequency *ω*. This equation is valid both for linear and nonlinear waves and allows us to include external power sources, denoted by *a*(*x*,*e*,*ω*), representing the amplification by outer hair cells.

The model differential equation for the *steady state situation* can now be obtained in the following way. We first consider two points *x*_1_(*t*) and *x*_2_(*t*) moving with group velocity *v*(*x_i_*) = *v_G_*(*k*(*x_i_*,*ω*),*ω*), *i* = 1,2, where *k*(*x*,*ω*) denotes the location-dependent wave number. The energy contained between these two points is
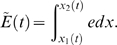
(14)From Equation 13 we obtain for the change of *E*∼ the expression
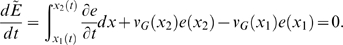
(15)Consider the case where *v_G_*(*x*_2_)*e*(*x*_2_)−*v_G_*(*x*_1_)*e*(*x*_1_)>0, i.e., if more energy leaves the interval [*x*_1_(*t*),*x*_2_(*t*)] than enters it. From this, it follows that the first term on the right hand side, and therefore ∂*e*/∂*t*, must be negative. Work done by the surrounding—by an amplification mechanism—would therefore be expressed by a negative contribution −*a*(*x*,*e*,*ω*) (work done by unit length). On the other hand, dissipative losses would appear as positive contributions. From this, we conclude that
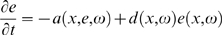
(16)holds, where *d*(*x*,*ω*) denotes viscous dissipation. Inserting this expression into the energy balance Equation 13, we obtain the *cochlea model differential equation* [3]

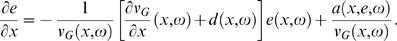
(17)Traditional cochlear models generally lead to partial differential equations, involving often detailed assumptions about geometry and forces within the cochlear canal. In contrast, our energy-based approach leads to an ordinary differential equation. This allows us, e.g., to concentrate on basic assumptions about the nature of the active amplifier only. In the absence of active amplification (*a*(*e*,*x*,*ω*)≡0), as considered here, Equation 17 is readily integrated to
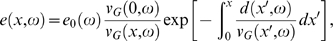
(18)where *e*_0_(*ω*) and *v_G_*(0,*ω*) denote the initial condition and the group velocity at stapes, respectively.

For the evaluation of the the traveling wave amplitude *A*(*x*,*ω*), we assume a linear fluid, *v*(*x*,*ω*) = *ωA*(*x*,*ω*). In this case, the equipartition principle implies that *e*(*x*,*ω*) 2*e*_pot_(*x*,*ω*) = 2*e*_kin_(*x*,*ω*). The density of the potential energy is determined by the transversal stiffness *E*(*x*) and the BM displacement *ζ*(*t*). In linear approximation, by Hooke's law we have
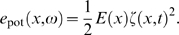
For sinusoidal displacements *ζ*(*x*,*t*) = *A* sin(*ωt*+*θ*) we obtain a time average of
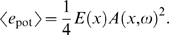
The energy density *e*(*x*,*ω*) and the BM stiffness *E*(*x*) are therefore related by
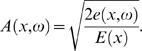
(19)Inserting Equation 18 into the last equation and denoting the displacement amplitude at stapes by *A*_0_, the passive traveling wave amplitude is described by
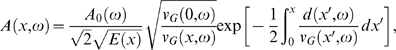
(20)where *A*_0_(*ω*) : = *ωA*_0_. In order to render this expression computationally seizable, we need to specify the functions *E*(*x*), *v_G_*(*x*,*ω*), and *d*(*x*,*ω*) and the remaining parameters.

For the evaluation of these functions, we proceed similarly to what is explained to more detail in [3]. From a two-dimensional surface-wave analogy of the cochlea [16], the dispersion relation
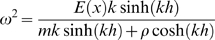
can be derived. The group velocity *v_G_* of the travelling wave then obtains the expression
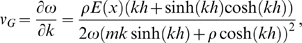
(21)where *ρ* is the cochlear fluid density, *m* the BM mass density, and *h* the diameter of the cochlear canal. For convenience we note that the dispersion relation can be written in the form
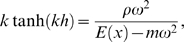
(22)which shows that the dispersion relation defines a locus *x_cc_*(*ω*) where the wave number *k* and, as will be seen in Equation 23, the dissipation diverges.

Whereas for the investigation in mind we can dispose of the active amplification contribution *a*(*x*,*e*,*ω*), the dissipation rate *d*(*x*,*ω*) is needed. The dissipation is composed from different sources, among them as the most prominent ones the internal friction *d_I_* = 4*νk* ^2^ (as originally derived by Stokes), where *ν* is the fluid kinematic viscosity and the friction between the moving fluid and the vibrating BM surface, which equals [9] 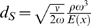. As a result, we are left with a dissipation rate of
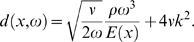
(23)A similar equation was already obtained by Lighthill [17]. By inspection of Equations 20–23, it is easily seen that the locus of maximal traveling wave amplitude, associated with a frequency *ω*, is related in a 1-to-1 way with a characteristic place *x_c_*(*ω*)≲*x_cc_*(*ω*). This defines the TM.

Numerical evaluation of the TM from Equation 20 also requires the values of the remaining constants and the form of *E*(*x*). Typical values at natural conditions (‘natural parameters’) are *ν* = 0.01 m^2^ s^−1^, *ρ* = 2000 kg m^−3^, *m* = 0.5 kg m^−2^, *h* = 10^−3^ m. The transversal stiffness, finally, has the form *E*(*x*) = *E*_0_ e^−*αx*^, with the decay exponent *α* = 3×10^4^ m^−1^. The surface tension contribution *T*(*x*) can be incorporated by means of a modified transversal stiffness *E*(*x*) = *E*_0_ e^−*αx*^+*k*(*x*)^2^ *T*(*x*) = (1+*Fk*(*x*)^2^)*E*_0_ e^−*αx*^, with the proportionality constant *F* = 10^−9^ m^2^, see [3]. At natural parameters, this reveals a linear semilogarithmic behavior of the form
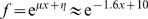
(24)(cf. Figures 3 and 4), where the obtained values result from a least-squares-fit (mean absolute error 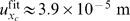, mean absolute error deviation 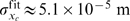).

### Short-Wave Approximation

In order to estimate the effects introduced by changed biophysical parameters, the space derivative of the travelling wave amplitude, 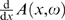 needs to be evaluated. Below, we consider our model without the surface tension term *T*(*x*) [3],[9]. In principle, it would be desirable to include this contribution [3], for which one would have to substitute in the dispersion relation and in the expression obtained for the group velocity the term *E*(*x*) by *E*(*x*)+*k* ^2^ *T*(*x*), where *k* is the wave number. On first view this looks like a simple modification; for the analytical expression of the response peak, the extra term, however, is a major obstacle, since *k* ^2^ changes in a complex manner in the neighborhood of the peak. Whereas an analytical treatment appears nearly impossible, the problem stated in this form is accessible to numerical approaches, via Equation 20. Since we will be interested in the location of the response peak alone, we expect that the surface tension can be neglected. Figures 3 and 4, where the numerical approach and the analytical approximation are compared, demonstrate that this is indeed the case. For the model without surface tension, from Equation 20 it emerges that
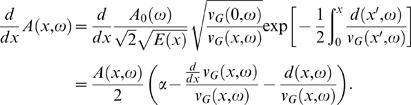
With *A*(*x_c_*,*ω*)≠0∀*x_c_*(*ω*), at all characteristic places *x* = *x_c_*(*ω*) the relation
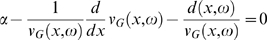
(25)has to be fulfilled. This equation, however, cannot be solved analytically.

Since we are interested in the close neighborhood of the peak only, where *k* becomes exceedingly large, so that *kh*≫1 (the short-wave limit), we obtain an approximation that is sufficiently accurate and manageable at the same time. In the short-wave limit, we obtain the relation
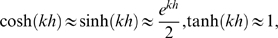
(26)which is valid in the vicinity of the characteristic place *x_c_*(*ω*), i.e., the place of interest. Insertion of Equation 26 into Equations 21 and 22 yields
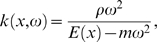
(27)

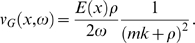
(28)By inserting Equation 27 in Equation 28, we obtain
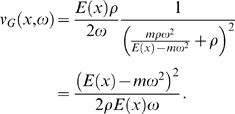
(29)By using *E*(*x*) = *E*_0_ exp(−*αx*) in Equation 29, we obtain for the space derivative of *v_G_*(*x*,*ω*)
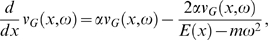
(30)from which it follows that
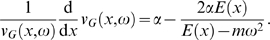
(31)Inserting Equation 27 in Equation 23 leads to
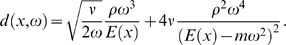
(32)Dividing the dissipation rate *d*(*x*,*ω*) by the group velocity *v_G_*(*x*,*ω*), Equation 29, we obtain
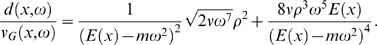
(33)Using Equations 31 and 33 in Equation 25 leads to
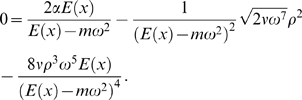
(34)Multiplication of this equation by (*E*(*x*)−*mω* ^2^)^4^ for convenience, and remembering that we required *x* = *x_c_*(*ω*), we arrive at the desired description of the impact of parameter variations to the TM,
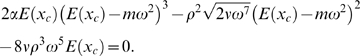
(35)We are unaware that with classical, not explicitly energy-based approaches [18],[19], a similar characterization could be achieved.

### Proof of the Theorem

Whereas Equation 35 is strongly nonlinear in the space coordinate *x*, the effects by variations of *m*, *ν*, and *ρ* on the TM are sufficiently simple to be discussed qualitatively as follows: Using the substitution Δ : = *E*(*x_c_*)−*mω* ^2^, for *x*≤*x_c_*(*ω*), Equation 35 can be written as
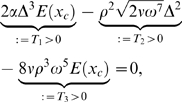
where the transversal stiffness *E*(*x_c_*), *mω* ^2^, and *E*(*x_c_*)−*mω* ^2^ are positive (otherwise for any value of *x*, Equation 35 would not hold). *E*(*x_c_*) and *mω* ^2^ are of the same order of magnitude. For Equation 35 to hold, *T*_1_ needs to balance the two subtractive terms *T*_2_ and *T*_3_. Due to the different powers associated with the parameters in *T*_1,2,3_, the TM is affected more by changes in *m* and *ρ* than by changes in *ν*. Although some general features of changed biophysical parameters might be extrapolated from various arguments, the mathematical demonstration of such properties as given in the following theorem, as well as the detailed statements provided by Part II of the theorem, appears to be novel.

#### Proof: Part I

For *ρ* and *ν* consider
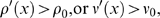
respectively, where index 0 indicates an original, constant, parameter value. As is easy to see,
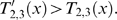
Since 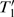 needs to compensate 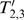, it follows that
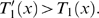
With *E*(*x*) = *E*_0_ e^−*αx*^, we obtain
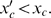
In the case of <, the inequalities simply invert.

For *m* we similarly assume
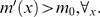
It then follows that
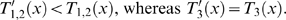
Δ^3^ in *T*_1_ reacts stronger upon changes in *m* than Δ^2^ in *T*_2_. Therefore, we obtain
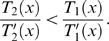
Hence, we can compensate the increase in *T*_2_ and *T*_3_ by an increase of *T*_1_. Such an increase, however, again implies
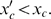
In the case of *m*′(*x*)<*m*_0_, the inequalities invert.

#### Proof: Part II

Inserting into Equation 35 a modified transversal stiffness *E′*(x′) = *E*_0_′ e^−*α*′*g*(*x*′)^ (where *g*(*x*′) is invertible but otherwise arbitrary), we obtain
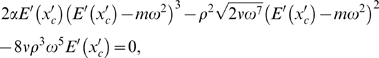
(36)where *x_c_*′(*ω*) is the new characteristic place. A comparison of Equation 35 with Equation 36, together with the uniqueness of the biologically reasonable solution of Equation 35, yields
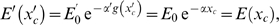
which relates *x_c_*′ to *x_c_* in a unique way. Due to the invertibility of *g*(*x*), *x_c_*′ can be expressed as
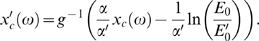
Taking into account Equation 24, it follows that
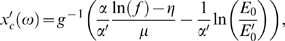
(37)with *η* as in Equation 24.

The second part of the theorem is new and can be seen as the direct benefit of our energy-based description. Equation 35 predicts numerically the effects generated by variations in the transversal stiffness *E*(*x*). TM obtained from variations of BM transversal stiffness, evaluated numerically from Equation 20 and predicted analytically by Equation 37, are compared in Figure 4. Taking as the reference the numerical evaluations of the TM from Equation 20, the approximation Equation 35 predicts the effects on the TM up to a numerical accuracy of ∼1.5×10^−2^ cm. The figures reveal that changes in the initial transversal stiffness *E*_0_ lead to a shift of the TM parallel to the original TM (Figure 4a). Variations in the decay constant *α* result in a rotation of the TM around the intersection of the original TM with the frequency axis (Figure 4b). Alterations of the space-dependence of the exponent, i.e., *x*→*g*(*x*), result in a non-linear space-dependence of the TM in a semilogarithmic plot (Figure 3).
